# A rare case of bilateral subclavian artery agenesis

**DOI:** 10.1016/j.jvscit.2023.101353

**Published:** 2023-10-24

**Authors:** Andria N. Li, Gulrez Mahmood, Clifford L. Garrard

**Affiliations:** a Vanderbilt University School of Medicine, Nashville, TN; b CVT Surgical Center, Baton Rouge, LA; c Division of Vascular Surgery, Vanderbilt University Medical Center, Nashville, TN

**Keywords:** Subclavian aneurysm, Arterial agenesis, Vascular medicine

## Abstract

Arterial agenesis is a rare condition and has been reported to affect the internal carotid artery, common carotid artery, and pulmonary artery. However, to the best of our knowledge, it has not yet been reported to affect the subclavian arteries. We present a case of asymptomatic bilateral subclavian artery agenesis and left subclavian artery aneurysm. This patient's abnormal vasculature was found incidentally. Despite being asymptomatic, repair of the aneurysm via vertebral transposition and ligation of the subclavian artery was performed to prevent eventual thrombosis, emboli, and stroke.

## Case report

**Fig 1:**
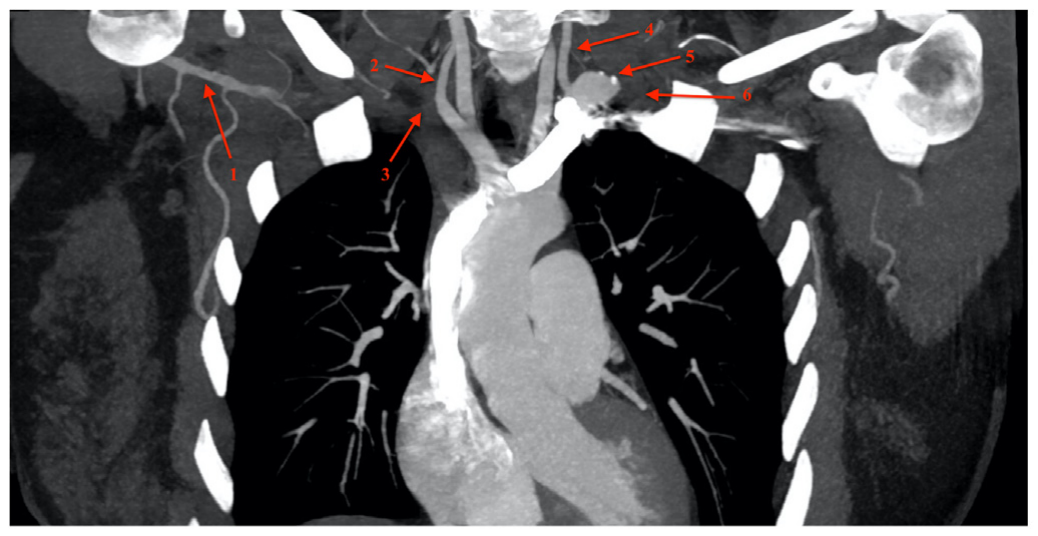
Computed tomography scan showing reconstitution of distal right axillary artery (*1*), right vertebral artery (*2*), expected course of the right subclavian artery (*3*), left vertebral artery (*4*), left subclavian artery aneurysm (*5*), and expected course of left subclavian artery (*6*).

**Fig 2:**
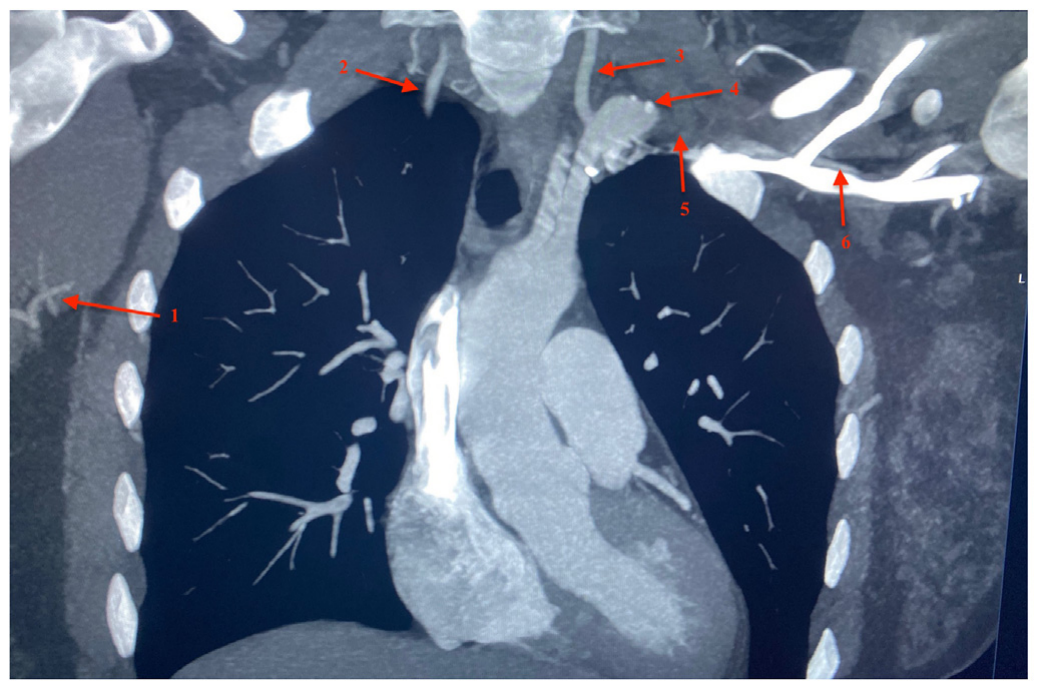
Computed tomography scan showing branches of distal right axillary artery (*1*), right vertebral artery (*2*), left vertebral artery (*3*), left subclavian artery aneurysm (*4*), expected course of left subclavian artery (*5*), and reconstitution of left axillary artery (*6*).

A 30-year-old woman presented to an outside hospital emergency department with symptoms of oropharyngeal swelling and dysphagia. As part of a workup for her symptoms, a computed tomography scan of the head and neck was obtained. Although it presented no explanation for her presenting symptoms, it demonstrated an incidental finding of bilateral subclavian artery agenesis (Fig 1). On the left, the subclavian artery terminated into a 2-cm aneurysm with mural thrombus just proximal to the left vertebral artery. Agenesis of the right subclavian and proximal axillary arteries was also present (Fig 2). The left and right axillary arteries reconstituted distally from collateral branches. The anatomy of the thoracic outlet appeared normal. Full body images were not performed because the patient was asymptomatic from these vascular anomalies. The patient provided written informed consent for the report of her case details and imaging studies.

The patient's symptoms of dysphagia resolved during her emergency department visit, and she later presented to our vascular clinic on an outpatient basis. On review of systems, she denied any history of activity-associated upper extremity ischemic symptoms, traumatic injuries to the upper extremity, hyper-flexibility, joint laxity, or symptoms of posterior circulation ischemia or stroke. She had no abnormalities in arm development. Her family history was negative for aneurysmal disease or any genetic connective tissue disorder. The patient denied any tobacco use. On physical examination, the patient had diminished but palpable radial pulses bilaterally. Given the mural thrombus and the proximity of the vertebral artery to the left subclavian aneurysm, there was concern about potential embolization to the vertebral artery. Therefore, resection of the aneurysm with transposition of the left vertebral artery to left common carotid artery was recommended to the patient.

The operation was performed under general anesthesia. A single incision was made on the left neck just above the clavicle. The sternal head of the sternocleidomastoid muscle was transected, and the carotid artery sheath was entered. The thoracic duct was identified and protected. The subclavian aneurysm was easily identified just proximal to the vertebral artery. A large vascular clip was placed on the proximal subclavian artery, and the aneurysm was resected. The vertebral artery was transected with a Carrel patch, and this was transposed onto the common carotid artery. The vertebral artery was unclamped, followed by unclamping of the common carotid artery. Doppler ultrasound was used to verify blood flow through the vertebral and carotid arteries.

The patient was discharged uneventfully. The 6-month follow-up carotid duplex ultrasound verified antegrade flow in both vertebral arteries. The bilateral brachial pressures were 112 mm Hg on the left and 119 mm Hg on the right. She remained asymptomatic. The patient has had no complications from the surgical intervention, and follow-up is scheduled for 1 year after surgery.

The patient was sent for a genetic evaluation for connective tissue disorders. This testing revealed three variants of unknown significance in DCHS1, MYH11, and SLC2A10. DCHS1 is associated with autosomal dominant mitral valve prolapse, MYH11 is associated with autosomal dominant thoracic aortic aneurysms and dissections, and SLC2A10 is associated with autosomal recessive arterial tortuosity syndrome. The patient has been referred to cardiology and vascular medicine. Her family members have also been recommended to undergo genetic testing.

## Discussion

**Fig 3:**
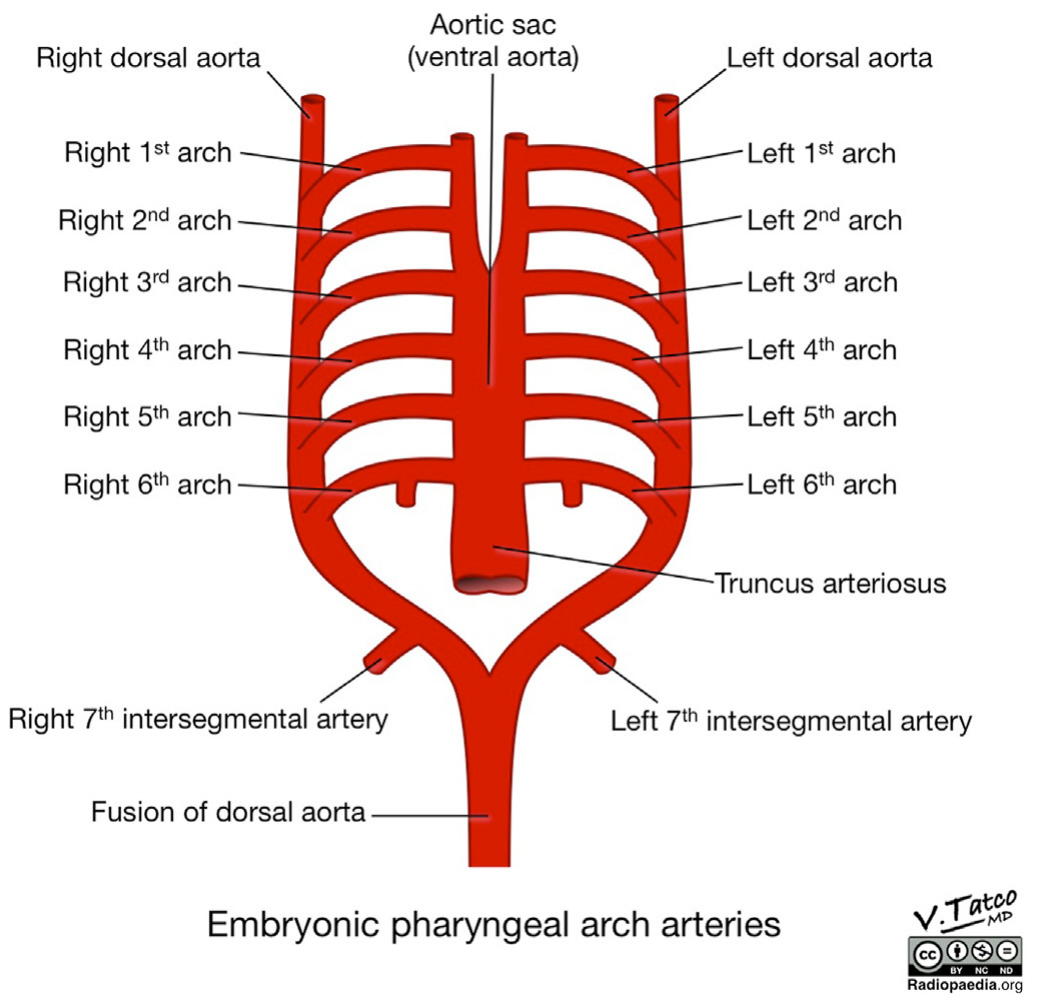
Illustration of embryonic pharyngeal arch arteries.

Although the literature has described aberrant subclavian arteries,^1^^,^^2^ isolated subclavian arteries,^3^^,^^4^ the absence of brachial arteries,^5^ agenesis of the internal carotid artery,^6^ and unilateral subclavian artery agenesis,^7^^,^^8^ bilateral subclavian artery agenesis is rare and, to the best of our knowledge, has not been reported. The left subclavian artery arises entirely from the left seventh intersegmental artery, and the right subclavian artery arises from the right fourth aortic arch proximally and the right seventh intersegmental artery distally (Fig 3).^9^^,^^10^ The exact cause of agenesis is not known; however, possible etiologies include a genetic abnormality or localized hypoxia during embryogenesis. Subclavian artery aneurysms are rare, and manifestations can range from asymptomatic to upper extremity activity-associated ischemic symptoms, ischemic ulcers, digital gangrene, and neuropathy from brachial plexus compression.^11^ Proximal subclavian artery aneurysms are typically due to atherosclerosis, thoracic outlet syndrome, trauma, or connective tissue disorders such as Marfan syndrome. To the best of our knowledge, there have not been any reports of a subclavian artery aneurysm related to subclavian artery agenesis.

Courtesy of Vincent Tatco, Radiopaedia.org, rID: 52,193.

The patient was recommended surgical intervention due to concerns of potential embolization from mural thrombus to the posterior circulation, given the proximity of the vertebral artery to the aneurysm on the left subclavian artery. The proximal subclavian artery was ligated with a large metal clip, similar to our technique of carotid subclavian transposition. Additional endovascular plug placement from within the aorta was not thought to be necessary.

## Disclosures

None.
